# Semantic Segmentation of Surface Cracks in Urban Comprehensive Pipe Galleries Based on Global Attention

**DOI:** 10.3390/s24031005

**Published:** 2024-02-04

**Authors:** Yuan Zhou, Zhiyu Yang, Xiaofeng Bai, Chengwei Li, Shoubin Wang, Guili Peng, Guodong Li, Qinghua Wang, Huailei Chang

**Affiliations:** 1 School of Instrument Science and Engineering, Harbin Institute of Technology, Harbin 150001, China; yuanzhou277@163.com (Y.Z.); 18101365772@163.com (X.B.); 2 School of Control and Mechanical, Tianjin Chengjian University, Tianjin 300384, China; yzy666333@163.com; 3 STECOL Corporation, Power Construction Corporation of China, Tianjin 300384, China; vzym616@163.com (G.L.); vciw709@163.com (Q.W.); hvoi557@163.com (H.C.)

**Keywords:** crack, semantic segmentation, attention model, loss function, GA-SegNet

## Abstract

Cracks inside urban underground comprehensive pipe galleries are small and their characteristics are not obvious. Due to low lighting and large shadow areas, the differentiation between the cracks and background in an image is low. Most current semantic segmentation methods focus on overall segmentation and have a large perceptual range. However, for urban underground comprehensive pipe gallery crack segmentation tasks, it is difficult to pay attention to the detailed features of local edges to obtain accurate segmentation results. A Global Attention Segmentation Network (GA-SegNet) is proposed in this paper. The GA-SegNet is designed to perform semantic segmentation by incorporating global attention mechanisms. In order to perform precise pixel classification in the image, a residual separable convolution attention model is employed in an encoder to extract features at multiple scales. A global attention upsample model (GAM) is utilized in a decoder to enhance the connection between shallow-level features and deep abstract features, which could increase the attention of the network towards small cracks. By employing a balanced loss function, the contribution of crack pixels is increased while reducing the focus on background pixels in the overall loss. This approach aims to improve the segmentation accuracy of cracks. The comparative experimental results with other classic models show that the GA SegNet model proposed in this study has better segmentation performance and multiple evaluation indicators, and has advantages in segmentation accuracy and efficiency.

## 1. Introduction

Urban underground comprehensive pipe corridors have been widely applied in various municipal and transportation projects, serving as an important solution to urban underground pipeline issues. The advantages of urban underground comprehensive pipe corridors lie in their ability to consolidate various pipelines into a single underground space, facilitating daily maintenance and management by relevant personnel while avoiding frequent road disruptions. This ensures the integrity of the road surface and the durability of the pipelines, reducing the impact on normal traffic and residents’ daily lives. Additionally, it also reduces maintenance costs for roads and various types of pipelines.

Urban underground comprehensive pipe corridors that were constructed earlier have entered the maintenance period, while newly built corridors also face potential risks such as deformation of the tunnel structure. The geological conditions in the underground environment are highly complex. During long-term usage, underground pipe corridors are susceptible to various factors such as earthquakes, ground subsidence, and soil moisture. This could lead to internal wall cracks and other defects, resulting in issues like water leakage and collapse, which compromise the structural safety of the corridors. The presence of these cracks not only leads to resource wastage but also poses significant safety hazards. They could disrupt the normal operation of the city and threaten personal, material, and other aspects of residents’ safety. Therefore, it is crucial to conduct periodic inspections and maintenance of urban underground comprehensive pipe corridors, including the detection and repair of cracks and other defects, in a timely and effective manner.

Detection methods based on computer vision technology have been widely applied in the field of road, bridge, and tunnel defect detection. These methods are characterized by their efficiency and comprehensiveness and have become the primary means of detection. This innovative technology utilizes advanced image processing and pattern recognition algorithms to analyze and identify various defects with high precision and speed in a highly automated manner. It greatly improves the accuracy and efficiency of detection, providing strong technical support for ensuring the safety of transportation and buildings.

Traditional computer vision technology in the field of crack detection mainly relies on digital image processing techniques. It involves manually discerning features and utilizing various feature patterns such as frequency, edges, orientation gradients, grayscale, texture, and entropy, as well as designing certain feature constraints to accomplish identification. A novel method for road crack detection was proposed by Ying L et al. [1]. A road surface image was segmented into multiple small regions, and a wavelet transform-based algorithm was utilized to connect the crack areas and extract the linear features of surface cracks. A custom image processing algorithm was designed by Xu B et al. in [2] for road crack detection. This algorithm divides the image into crack elements or non-crack elements based on local features, comparing the crack seeds with their adjacent regions to verify the category, and after multiple verifications, determining the seed cluster as the actual crack. Shi et al. [3] proposed a road crack detection method, which introduces integral channel features for crack feature extraction and utilizes a random forest classifier to mine structured information, improving detection accuracy. Salman et al. [4] presented a crack detection method based on Gabor filtering. High-pass Gabor filters were employed to detect cracks in different directions. In [5], Sobel filtering was applied to remove noise from grayscale images, and crack detection was performed by the OTSU method. This method demonstrates good performance in detecting small cracks. H. Oliveira and P. L. Correia [6] proposed a new framework for the automated detection and classification of cracks in survey images obtained during high-speed driving. Sun L et al. [7] introduced the weighted neighborhood pixel method, which uses local thresholding and shape filtering with eccentricity parameters to enhance candidate cracks. It has the characteristics of accuracy, speed, robustness, and suitability for online road condition assessment. An automated method of classification and segmentation of asphalt pavement cracks was proposed by Y. Sari et al. in [8]. The classification method of the support vector machine (SVM) algorithm and the segmentation method of the OTSU algorithm were employed to classify the asphalt pavement cracks.

Traditional image segmentation algorithms typically have high time complexity and weak generalization ability due to artificially designed features. In recent years, deep learning has emerged as a dominant research direction in the field of computer vision, yielding numerous achievements in areas such as object detection, autonomous driving, and natural language processing. Deep learning, based on artificial neural networks, enables the automatic and efficient extraction of valuable information from large-scale data, significantly enhancing learning efficiency and enabling the resolution of more complex problems. With further advancements in deep learning research, algorithms that integrate deep learning and convolutional neural networks have achieved superior performance in the field of crack detection. Xiang et al. [9] constructed a crack recognition network using an encoder–decoder structure and employed a pyramid module to capture the contextual information of complex crack images from a global perspective. Wang et al. [10] proposed a road crack detection method based on pyramid convolution and a boundary enhancement network, which extracts features at multiple scales and further processes crack features through a boundary refinement module and depth monitoring module. It can accurately segment a complete crack area and sharpen its boundaries. Protopapadakis et al. [11] introduced a combination of deep convolutional neural networks and domain-specific heuristic post-processing techniques to fundamentally select effective features and complete crack detection tasks faster. The Enhanced Chicken Swarm Algorithm (ECSA) was utilized in [12] by Yu et al. to optimize the parameters of deep convolutional neural networks, improving the generalization ability of the crack detection model. Yue Pan et al. [13] proposed a spatial channel-wise network for pixel-level crack segmentation. This network fully exploits spatial and channel dependencies by adaptively integrating local features through self-attention mechanisms, enhancing the segmentation performance of the network. Knig J et al. [14] proposed a decoder part for an encoder–decoder-based deep learning architecture for semantic segmentation. This method introduced a previously unused technique in the field of surface crack segmentation: test time augmentation for generating results, which enables obtaining state-of-the-art performance across all datasets. X. Sun et al. [15] adopted and enhanced DeepLabv3+ and proposed a multi-scale preservation module in the decoder to generate attention masks and dynamically allocate weights between high-level and low-level feature maps, effectively helping the model better integrate multi-scale features and generate more accurate road crack segmentation results. A new semantic translational representation network (STRNet) was proposed in [16] for the real-time segmentation of pixel-level cracks in complex scenes. A new encoder–decoder segmentation network, CycleADC-Net, was introduced by Yidan Yan et al. in [17], which opened up a new idea for crack image detection under low light conditions. A lightweight remote sensing object detection model called Attention and Multi-Scale Feature Fusion Lightweight YOLO is proposed by Peng et al. in [18], which could improve the accuracy of the network. Chu, H. et al. [19] proposed a multi-scale feature fusion network with an attentional mechanism called Tiny-Crack-Net (TCN), which utilized an improved residual network to capture the local features of tiny cracks. The effectiveness and robustness of the “Tiny-Crack-Net” were validated with field test results.

In recent years, semantic segmentation algorithms based on attention mechanisms have received increasing attention from both academia and industry. Due to the fact that attention mechanisms can simplify feature extraction methods, the performance of methods that introduce attention mechanisms exceeds that of most fully convolutional semantic segmentation methods. Recent research has extensively built their methods based on this idea. The attention mechanism has been proven to be effective in image semantic segmentation tasks. Rehman et al. [20] proposed a new encoder–decoder architecture for effectively segmenting brain tumor regions, which emphasizes and restores the segmentation output in the extracted feature maps by introducing an attention gate module. Chen et al. [21] proposed a novel transformer-based attention-guided network called TransAttUnet, in which multi-level-guided attention and a multi-scale skip connection are designed to jointly enhance the performance of semantical segmentation architecture. Aghdam et al. [22] proposed an attention-based Swin U-Net extension for medical image segmentation to improve the classical cascade operation in skip connection paths by introducing attention mechanisms. Coquenet et al. [23] proposed an end-to-end non-segmented architecture for handwritten-document recognition tasks based on an attention mechanism: Document Attention Network, which achieved good recognition results.

In the task of crack detection in urban underground utility tunnels, the basic step is to determine the presence of cracks in the images. Additionally, the model needs to extract the semantic feature information of the cracks and perform segmentation of the crack regions. The cracks inside urban underground utility tunnels are often small and lack prominent features. Furthermore, factors like low illumination and large shadow areas lead to low differentiation between cracks and the background in the images.

Therefore, this study proposes a Global Attention-based Semantic Segmentation Network (GA-SegNet) to address the aforementioned issues. The main contributions of this work are summarized as follows.

A. A new residual separable convolutional attention model is proposed as an encoder. By using depth separable convolution and a residual attention mechanism, more efficient crack feature extraction is achieved. A pyramid structure is used to extract features at multiple scales, achieving the accurate classification of image pixels.

B. In order to adapt to multi-scale features while reducing computational complexity, the decoder uses a global attention upsampling model to enhance the feature connection between the encoder and decoder, quickly and effectively adapt to feature mapping at different scales, achieve simple and efficient image reconstruction, improve the multi-scale feature extraction ability of the network, and improve the segmentation effect for small cracks.

C. By using a balanced loss function, the contribution of crack pixels is increased in the total loss, while the attention of background pixels is reduced, bringing significant gains to the crack segmentation task.

The organizational structure of this article is as follows: Section 2 introduces the background of the research. Section 3 proposes a crack semantic segmentation network based on global attention and provides a detailed explanation of the structures and principles of the internal encoder and decoder. Section 4 conducts experimental analysis on the performance of the proposed semantic segmentation network and compares it with reference networks. Section 5 summarizes the article.

## 2. Related Works

Each pixel in an image carries its own information, such as color, texture, and spatial position, which collectively form the different elements in the image. Image semantic segmentation is a pixel-level classification method that involves categorizing pixels into different classes and then reconstructing the image based on the classification results. Traditional image segmentation algorithms rely on extracting low-level features to guide the segmentation process, but these methods often suffer from low accuracy.

**Figure 1:**
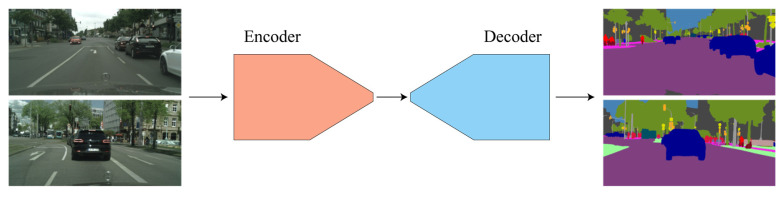
Semantic segmentation based on fully convolutional neural network.

The advancement of computer hardware and the improvement in GPU computing power have provided effective support for further research in semantic segmentation methods. Figure 1 depicts a semantic segmentation model based on fully convolutional neural networks, which has become the mainstream method in the field of semantic segmentation due to its superior feature extraction performance. Compared to traditional image segmentation methods, FCNs enable end-to-end, pixel-to-pixel segmentation algorithms, allowing for the extraction of higher-level semantic information from images and significantly improving segmentation accuracy. Researchers have proposed a series of classic segmentation networks based on fully convolutional neural networks. Long et al. [24] adapted contemporary classification networks into fully convolutional networks and transferred their learned representations by fine-tuning to the segmentation task. Badrinarayanan et al. [25] presented a novel and practical deep fully convolutional neural network architecture for semantic pixel-wise segmentation termed SegNet. The decoder of the network upsamples input feature maps at lower resolutions, eliminating the need for learning to improve sampling rates. Ronneberger et al. [26] proposed the U-Net network, which can be trained end-to-end from a very small number of images and has a fast network speed. For scene parsing tasks, Zhao et al. [27] proposed a pyramid scene parsing network that utilizes global context information through different region-based context aggregation, achieving good performance. Chen et al. proposed an approach to spatial pyramid pooling (ASPP) to robust segment objects at multiple scales, which addresses the task of using deep learning for semantic image segmentation [28,29,30,31]. These networks have had a significant impact on subsequent research in semantic segmentation.

However, most current semantic segmentation methods focus on global segmentation with a large receptive field. Nevertheless, in many application scenarios, the task is to segment small objects, requiring more attention to local edge details for more accurate segmentation results. Additionally, existing methods have increased model complexity while improving segmentation accuracy, necessitating the need to reduce model complexity and improve segmentation efficiency while maintaining accuracy. In the context of underground utility tunnels, cracks are often small and lack distinct features. Moreover, low illumination and large shadow areas further decrease the discrimination between cracks and the background in captured images. Based on the above analysis, this article focuses on the research of the encoder, decoder, and loss function, and builds a semantic segmentation network model to accomplish crack segmentation tasks in urban underground utility tunnels.

## 3. Global Attention-Based Semantic Segmentation Network for Cracks

### 3.1. The Overall Structure of the Semantic Segmentation Network

To address the issues of small and indistinct cracks in urban underground utility tunnels, as well as the low discrimination and imbalanced pixel distribution between cracks and the background in captured images, a Global Attention Segmentation Network (GA-SegNet) based on global attention is proposed in this article.

**Figure 2:**
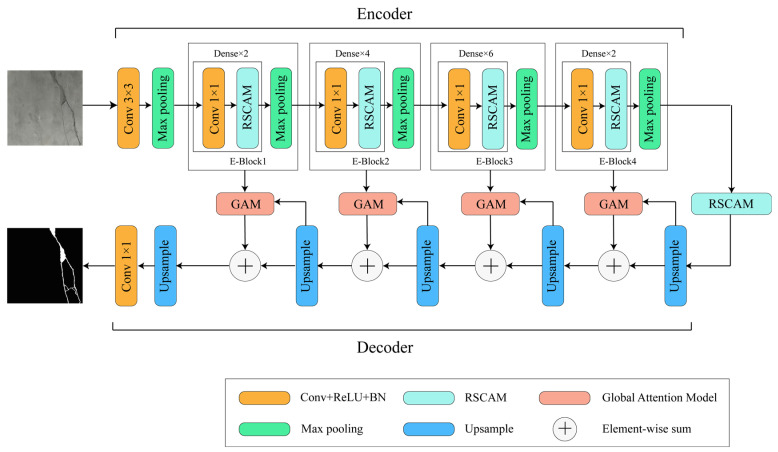
The overall structure diagram of GA-SegNet.

Figure 2 illustrates the overall structure of GA-SegNet, which consists of an encoder and a decoder. The encoder utilizes four residual separable convolution pyramid attention models as the backbone network to extract and classify pixel features in the image. The decoder part deviates from the classical symmetric structure and instead employs four Global Attention Modules (GAMs). These modules could quickly and effectively restore the details of the original image. The global semantic information obtained from high-level features in the decoder stage guides the weighted operations of low-level features. Additionally, an independent residual separable convolution attention model is embedded between the encoder and decoder to further integrate contextual information of the image and provide better pixel-level attention to high-level features in the decoder stage. The following are detailed introductions to each module.

### 3.2. Encoder

The main task of the encoder is pixel-level classification of the image and typically utilizing a convolutional neural network to assign initial class labels to each pixel. The encoder combines multiple convolutional and pooling layers hierarchically, allowing it to effectively capture local features in the image and progressively abstract high-level semantic information at multiple scales. It ultimately outputs a low-resolution image with labeled pixels, where each label represents a specific feature.

**Figure 3:**
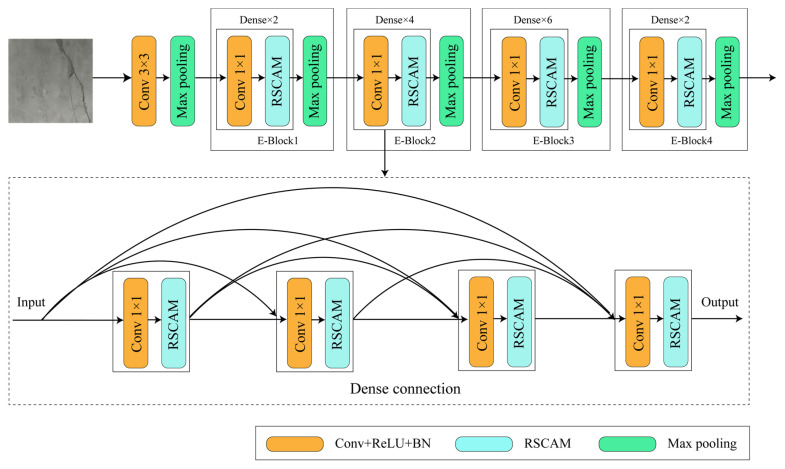
Encoder structure diagram.

As shown in Figure 3, the encoder consists of four E-blocks. The input image data undergo 3 × 3 convolution and max pooling operations for standardization and preprocessing. The other four E-blocks are composed of 1 × 1 convolutions, residual separable convolution attention models, and max pooling. They are also internally connected in a dense manner. The residual separable convolution attention models fuse feature information from multi-scale channels, enabling comprehensive capture of pixel-level semantic information in the image. Subsequently, a series of max pooling operations are applied to obtain low-dimensional feature information related to object edges, colors, and other characteristics.

#### Residual Separable Convolutional Pyramid Attention Modeling

Typically, a fully convolutional neural network encoder could utilize an image classification network. Nevertheless, the significant difference in pixel distribution between cracks and background in the image poses a challenge. It causes the encoder to be biased towards focusing on the features of background pixels during the training process. To address this issue, this study proposes a residual separable convolution attention model (RSCAM) as the baseline network. This model directs more attention towards the feature extraction of crack pixels.

**Figure 4:**
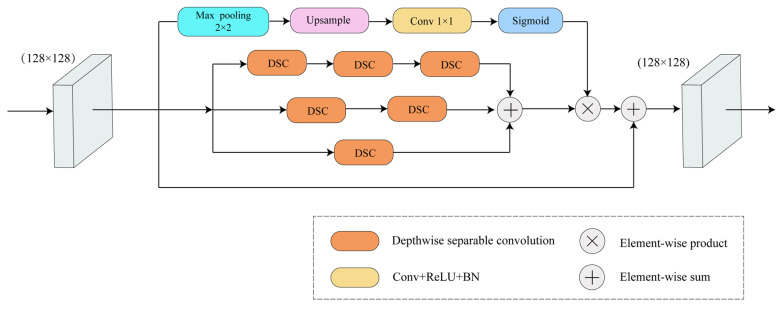
Residual separable convolutional pyramid attention model.

As shown in Figure 4, the model utilizes multiple depthwise separable convolutions [32] as the primary feature extractor. A residual attention mechanism is utilized to effectively reduce information loss and improve convergence speed during the stacking process. The model adopts a pyramid structure internally, where the input image passes through multiple depthwise separable convolution layers. The extracted features are then summed up, weighted with the soft mask branch of multi-scale features, and added to the original features to obtain the final output.

Depthwise separable convolution significantly reduces the number of model parameters by dividing the feature extraction process of conventional convolutions into two simpler steps: depthwise convolution and pointwise convolution. The computation formulas are as follows:

(1) $$DC\mathrm{onv}{\left(W,x\right)}_{\left(i,j\right)}=\sum_{a,b}^{A,B}W_{\left(a,b\right)}\cdot x_{\left(i+a,j+b\right)}$$

(2) $$PConv{\left(W,x\right)}_{\left(i,j\right)}=\sum_{t}^{T}W_{t}\cdot x_{\left(i,j\right)}$$

(3) $$Conv{\left(W_{p},W_{d},x\right)}_{\left(i,j\right)}=PConv{\left(W,x\right)}_{\left(i,j\right)}\left(W_{p},DC\mathrm{onv}{\left(W,x\right)}_{\left(i,j\right)}\right)$$

In the equations, $DC\mathrm{onv}\left(W,x\right)$ represents the channel-wise convolution process. $\;PConv\left(W,x\right)$ represents the pointwise convolution process. $\;Conv\left(W_{p},W_{d},x\right)$ represents the depthwise separable convolution process. x  represents the input feature. $\left(i,j\right)$ represents the coordinates of the output feature map. a,b,t  represents the size of the convolution kernel. W represents the convolution weight matrix.

### 3.3. Decoder

The function of the decoder is to process the low-dimensional feature information obtained from the encoder stage into high-dimensional feature information containing semantic and object classification-related information. Its essence is to restore the low-resolution image output from the encoder to the resolution of the original input image through deconvolution or upsampling operations. Finally, a classification layer is applied to accomplish pixel-level classification tasks. One of the representative early deep learning-based semantic segmentation networks is FCN (fully convolutional network). FCN modified the fully connected layers of image classification networks into convolutional layers but did not consider the relationships between pixels. Therefore, researchers started to consider fully utilizing the low-level information in the decoder and using it as guidance to help the high-level features recover image details. The most direct way is to add pathways between the encoder and decoder, such as SegNet, U-Net, DeepLab, and other networks.

**Figure 5:**
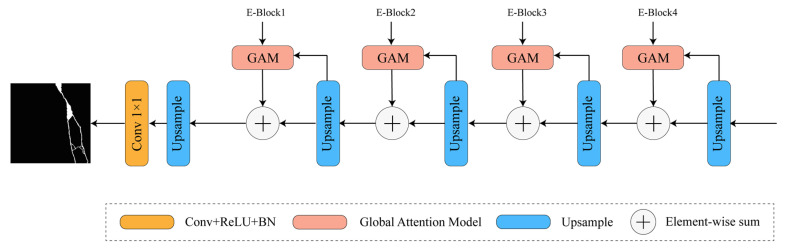
Decoder structure diagram.

Taking all factors into consideration, a global attention model was employed as the decoder in this study. By performing simpler computations, it weights the high-dimensional features onto the low-dimensional feature maps. This approach could adapt to features of different scales, reduce computational complexity, and simplify and enhance the efficiency of the image reconstruction process. The structure of the decoder is illustrated in Figure 5, consisting of four global attention upsampling modules. The high-dimensional features undergo multiple upsampling operations and are fused with the low-dimensional features through weighted fusion. Finally, a classification layer is applied to accomplish pixel-level classification tasks, resulting in a semantic segmentation map that is consistent in size with the original image.

**Figure 6:**
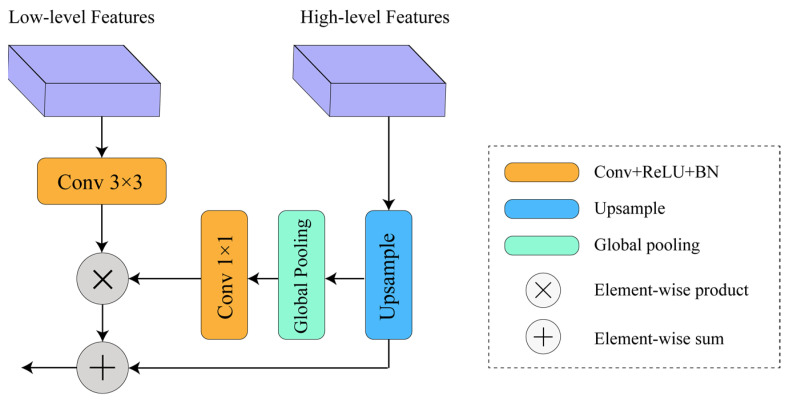
Global attention model.

The global attention model is depicted in Figure 6. Firstly, the low-level features extracted by the decoder undergo a 3 × 3 convolution operation to reduce the number of feature maps and obtain a more compact feature representation. The high-level features from the encoder are upsampled and then subjected to global average pooling to capture the global contextual semantic information of the image. Subsequently, 1 × 1 convolution, batch normalization, and non-linear transformation operations are performed to further refine the high-level features for better guidance in the weighted fusion with the low-level features. Finally, the upsampled high-level features are fused with the weighted low-level features, and successive upsampling operations are performed to restore the image’s resolution. The global attention upsampling model fully utilizes global contextual information and features of different scales, and combines them with the low-level information output by the decoder through weighted fusion, thereby improving the performance and efficiency of the decoder.

### 3.4. Loss Function

The purpose of a loss function is to evaluate the accuracy of a model by comparing its predicted results with the ground truth annotated images. In the task of image semantic segmentation, the choice of a loss function needs to be determined based on the characteristics of the task, such as the morphology, size, and distribution of the segmentation targets. Therefore, selecting the appropriate loss function could stimulate the learning process of the model, thereby improving the efficiency and accuracy of the model’s learning. In the case of urban underground comprehensive pipeline crack images, the proportion of pixels in the crack region is small compared to the background region, which presents a class imbalance issue.

Class imbalance is a common problem encountered in object detection and segmentation tasks. During data collection, it is difficult to effectively control the pixel proportions of different classes in the image content manually. This may result in a significant difference in the number of pixels for each class in the image. Therefore, achieving balance among the pixel quantities of different classes is a challenging task. When the number of background pixels in the image is much larger than the number of crack pixels, the influence of crack pixels on the loss function becomes very small. This situation leads to significantly higher accuracy in background segmentation compared to crack segmentation. Although data augmentation techniques could effectively improve the model’s overfitting resistance, their effectiveness is not significant when dealing with class imbalance issues. Therefore, optimizing the loss function could be employed to address the class imbalance problem by increasing the weight of crack pixels in the overall loss calculation, allowing the model to focus more on crack samples. The loss functions to address class imbalance are as follows.

Weighted cross entropy loss is a loss function that introduces weights for each class in the image to alleviate foreground-background class imbalance. The formula for weighted cross entropy loss is shown in Equations (4) and (5).

(4) $$w=\frac{n-n_{true}}{n}$$

(5) $$loss=-w\times y_{true}\log\left(y_{pred}\right)-\left(1-y_{ture}\right)\log\left(1-y_{pred}\right)$$

In this context, w represents the weight coefficient, n represents the total number of pixels, $n_{true}$ represents the actual number of segmented crack pixels, $y_{true}$ represents the label category of crack samples, and $y_{pred}$ represents the model’s prediction result.

Dice loss is a commonly utilized similarity evaluation function for binary classification tasks, which could be utilized to compare the similarity between two samples. Nevertheless, when the similarity approaches 1, the gradient of dice loss becomes very small, leading to the issue of gradient saturation, which makes it difficult for the model to update its parameters. Its formula is shown as (6):

(6) $$loss=1-\frac{2\times \left(X_{true}\cap X_{pred}\right)}{X_{true}+X_{pred}}$$

In this context, $\;X_{true\;}$ represents the real crack sample set, and $\;X_{pred}\;$ represents the sample set predicted to be cracks.

Focal loss assigns higher weights to difficult-to-classify samples and rare classes, allowing the model to pay more attention to these samples. By adjusting the weights of these samples, it could effectively improve the learning performance of the model on minority classes and difficult-to-classify samples. The formula is shown as (7):

(7) $$loss=-x\alpha {\left(1-p\right)}^{\gamma }\log\left(p\right)-\left(1-x\right)\left(1-\alpha \right)p^{\gamma }\log(1-p)$$

In this formula, x represents the sample label category, α represents the balance adjustment parameter for positive and negative samples, with a value range of $\left[0,1\right]$. p denotes the model’s predicted probability. γ represents the balance parameter for easy and difficult samples. This loss function reduces the weight of easy-to-classify samples by controlling parameter γ. The larger the value of γ, the greater the penalty on easy-to-classify samples.

### 3.5. Evaluation Metrics

This study belongs to the pixel-level semantic segmentation task, aiming to label and classify crack and background category pixels in input images. Therefore, in this article, commonly used metrics in segmentation tasks are employed to evaluate the performance of the model, including frames per second (FPS), floating point operations (FLOPs), pixel accuracy (PA), mean pixel accuracy (mPA), and mean intersection over union (mIoU).

FPS is used to evaluate the processing speed of a model on a given hardware and refers to the number of images that can be processed per second. FLOPs are used to measure the computational complexity of the model.

Pixel accuracy (PA) is utilized to represent the proportion of correctly segmented pixels by the model among the total number of pixels in the image. Its formula is shown as Equation (8):

(8) $$\;\mathrm{PA}=\frac{\sum_{i=0}^{k}p_{ii}}{\sum_{i=0}^{k}\sum_{j=0}^{k}p_{ij}}=\frac{TP+TN}{TP+TN+FP+FN}$$

Mean pixel accuracy (mPA) refers to the average segmentation accuracy of the network for crack and background pixels in the image. Compared to the pixel accuracy metric, mean pixel accuracy provides a more comprehensive reflection of the model’s performance on different categories and better balances the segmentation performance among different classes, thus providing a more reliable overall evaluation result. Its formula is shown as Equation (9):

(9) $$\mathrm{mPA}=\frac{1}{k+1}\sum_{i=0}^{k}\frac{p_{ii}}{\sum_{j=0}^{k}p_{ij}}$$

Mean intersection over union (mIoU) represents the average ratio of the intersection to the union of the number of pixels between the true labeled categories and predicted results for cracks and background in the image. This metric could indicate the similarity between the predicted results of all categories and the ground truth labeled image, as shown in Equation (10):

(10) $$mIoU=\frac{1}{k+1}\sum_{i=0}^{k}\frac{p_{ii}}{\sum_{j=0}^{k}p_{ij}+\sum_{j=0}^{k}p_{ji}-p_{ii}}$$

In this equation, k+1 represents k foreground classes and 1 background class, where k=1 for the segmentation task in this article. $p_{ii}$ represents the probability of predicting class i as class i. $p_{ij}$ represents the probability of predicting class i as class j. $p_{ji}$ represents the probability of predicting class j as class i.

## 4. Experiments and Analysis

### 4.1. Dataset

A. Crack500

**Figure 7:**
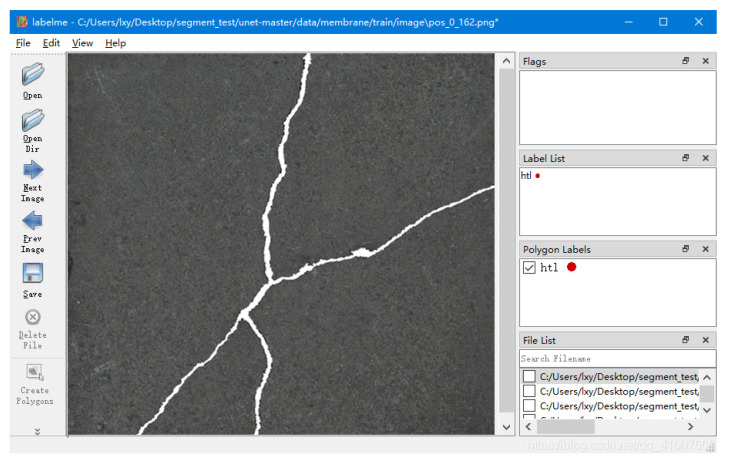
Labelme interface diagram.

The Crack500 dataset is a publicly available crack dataset that contains 476 crack images obtained through photos and image acquisition devices in real-life scenarios. By using this dataset, researchers can analyze and process crack images, continuously improving and optimizing crack detection algorithms, which is of great significance for ensuring the safety and maintenance of building structures. The image size is not uniform, with most being horizontal and a few being vertical. We cut the images in the dataset into fixed size images of 512 × 512. In order to avoid overfitting caused by small data volume, the dataset was expanded. Specifically, we expanded the dataset using horizontal flipping and random directional rotation operations, and divided the final dataset into training, validation, and testing sets in an 8:1:1 ratio. The final experimental data included 1144 images in the training set, 380 images in the validation set, and 380 images in the test set. We used Labelme 3.11.2 for annotation and generated corresponding annotation information files. Figure 7 shows the interface diagram of Labelme annotation software. Table 1 shows the detailed allocation table for the Crack500 dataset.

B. CCUIPC

**Figure 8:**
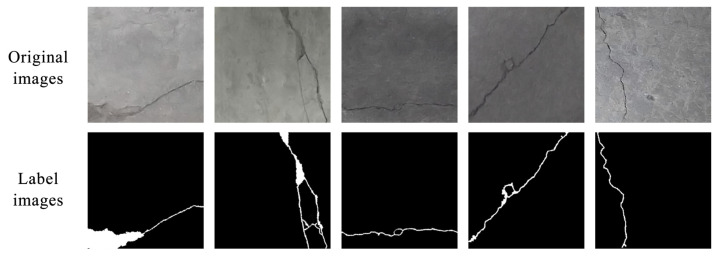
Original and annotated images of partial crack samples.

In order to further test the segmentation effect of the algorithm, a new concrete cracks in underground integrated pipeline corridors dataset (CCUIPC) was introduced. All images were collected from underground integrated pipelines in some cities in Tianjin, China, totaling 2000 images. We used Labelme 3.11.2 to label the 2000 crack images. The image size was adjusted to 512 × 512. Figure 8 shows the original and labeled images of cracks in some urban underground pipe corridors, where the white area is the labeled cracks. As shown in Table 2, according to the requirements of model training, the crack image dataset was divided in the ratio of 8:1:1, in which the training set contained 1600 crack images, and the validation set and the test set contained 200 crack images respectively.

All experiments in this study were conducted on a Windows 10 system environment. The NVIDIA GeForce GTX 1050 Ti GPU was utilized to support model training and inference, while the computer processor model was Intel Core i7-3770. Table 3 and Table 4 provide the relevant hardware and software configuration information.

### 4.2. Experimental Results and Analysis

In the training phase, the GA-SegNet model constructed in this article was optimized by the Adam optimizer. Due to the complexity of the network model and the storage capacity of the GPU, this study adjusted the batch size to 4 for training and set the total number of training batches to 180. The learning rate decay strategy was set to decrease by 10% every 20 batches, with $\beta_{1}=0.99$, $\beta_{2}=0.999$, and the weight decay set to 0.0001. Based on conventional learning rate setting methods and multiple debugging attempts, the initial learning rate for this experiment was set to 0.0001.

**Figure 9:**
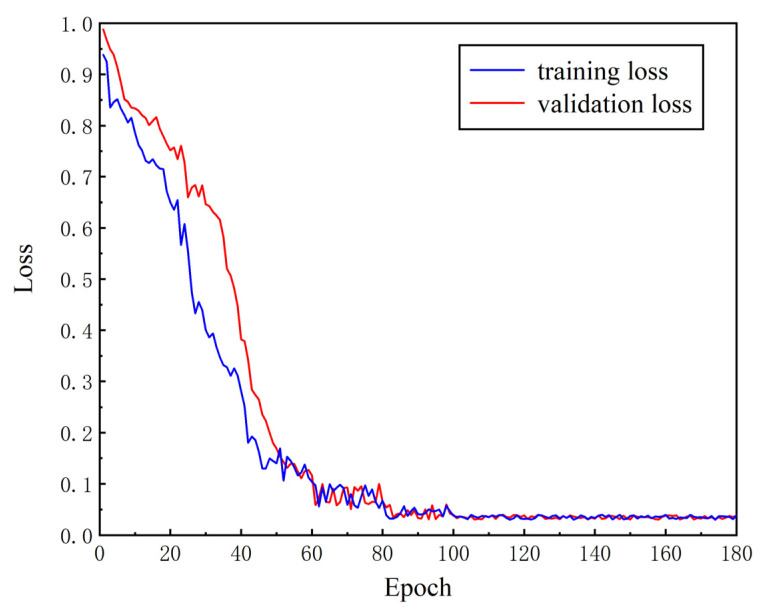
GA-SegNet model training and validation loss function.

Figure 9 shows the loss variation in the GA-SegNet model during training. It can be observed that the loss curves are almost completely overlapping, exhibiting relatively smooth changes, and overall showing a decreasing trend without overfitting. In the early stages, the learning rate is relatively high, resulting in fluctuations in the loss curves around the 20th and 40th training batches. Nevertheless, the loss curves stabilize thereafter and gradually converge. The loss function measures the difference between the predicted results of the model and the true results, reflecting the change in accuracy during the training process to some extent. Therefore, considering the comprehensive analysis, the GA-SegNet model performs best in terms of segmentation network model training when the initial learning rate is set to 0.0001.

### 4.3. Ablation Experiment

To evaluate the performance of our proposed GA-SegNet algorithm, we conducted ablation experiments to examine the impact of each improvement in the algorithm on its performance. The performance evaluation metrics include PA, mPA and mIoU.

Table 5 lists the experimental results obtained for GA-SegNet with various optimization measures. The ablation experiments used FCN as the baseline model. Aiming at the uneven distribution of pixel categories in the crack image samples of an urban underground comprehensive pipeline corridor, this paper selected three common loss functions for solving the category imbalance problem to conduct the training comparison experiments (weighted cross entropy loss, dice loss, and focal loss) and analyzed the improvement effect of different loss functions on the crack segmentation performance of the GA-SegNet model through the ablation experiments. “GAM” and “WCELoss” stand for global attention model and weighted cross entropy loss. Method (1) shows that when we used the residual separable convolutional attention model as the benchmark network, and when the model was also trained using the weighted cross entropy loss function, the PA, mPA and of the network were improved by 2.27% and 0.99%, respectively, compared to the benchmark, but the mIoU was slightly decreased by 1.2% compared to the benchmark. Method (2) shows that when the global attention model was used as the decoder and the model was trained using the weighted cross-entropy loss function, all the performance evaluation metrics of the network were improved, and method (3) shows that when the algorithm introduced both the residual separable convolutional attention model and the global attention model, all the performance evaluation metrics of the network were further improved compared to the above-mentioned methods, and the network’s PA, mPA and mIoU were improved by 4.24%, 1.88% and 1.78%, respectively, compared to the benchmark, which proves the effectiveness of the various improvements in the encoder and decoder in this paper.

Method (4) shows that the use of the dice loss function had a better effect on the model performance in the binary classification task, and the PA, mPA and mIoU of the network were improved by 1.66%, 3.4% and 0.75%, respectively, compared with method (3); method (5) shows that the model trained with focal loss function performed best in the test set, with a PA of 98.69%, mPA of 89.52%, and mIoU of 85.73%, which can significantly improve the segmentation performance of the GASN model for cracks. In this paper, an optimal combination of methods (method (5)) was chosen to ensure the best performance of the model.

### 4.4. Comparative Experiment

We compared our method with FCN, SegNet, U-Net, Deep Lab V3, and PSP Net in terms of model complexity and algorithm efficiency.

Table 6 presents the comparison results between the proposed method and the state-of-the-art methods mentioned above in terms of FPS and FLOPs. Compared with other algorithms, GA-SegNet achieved the fastest inference speed and the smallest model complexity (FPS: 47, FLOPs: 87.6 G). The inference speed was 11.4% faster than the fastest algorithm (PSP Net). The model complexity decreased by 7.3% compared to FCN, to only 30.6% of SegNet (286 G FLOP). The experimental results demonstrate the effectiveness of the residual separable convolutional pyramid attention model, significantly reducing model complexity while maintaining good inference speed, and meeting the real-time segmentation requirements of crack targets.

To validate the superiority of the proposed GA-SegNet model, comparative experiments on semantic segmentation are conducted with FCN, SegNet, U-Net, DeepLab V3, and PSPNet as reference models. Crack500 and CCUIPC datasets are utilized for training and testing, and the training strategies are continuously adjusted to achieve optimal segmentation performance.

**Figure 10:**
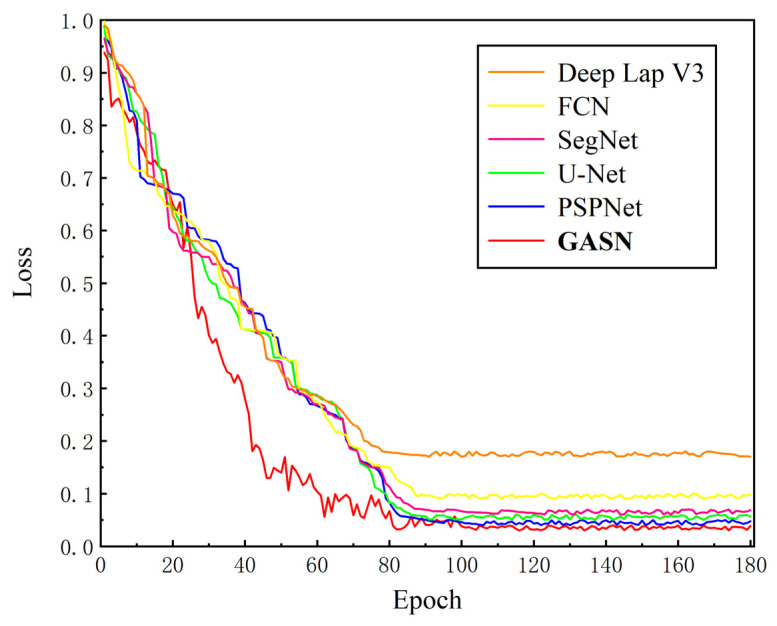
Change of loss curve in the training process of GA-SegNet and reference model.

The training loss curves of GA-SegNet and the reference models are shown in Figure 10. Among them, DeepLab V3 has the highest loss value, indicating the poorest training performance among the mentioned models. In comparison, U-Net and SegNet have similar training losses. PSPNet shows good training performance, with a final loss convergence value close to that of the proposed GA-SegNet network. Compared to the other models, the GA-SegNet network exhibits faster convergence of training loss and achieves the smallest stable loss value. This indicates that it could learn the discriminative criteria of pixels in images more quickly and achieve higher segmentation accuracy.

**Figure 11:**
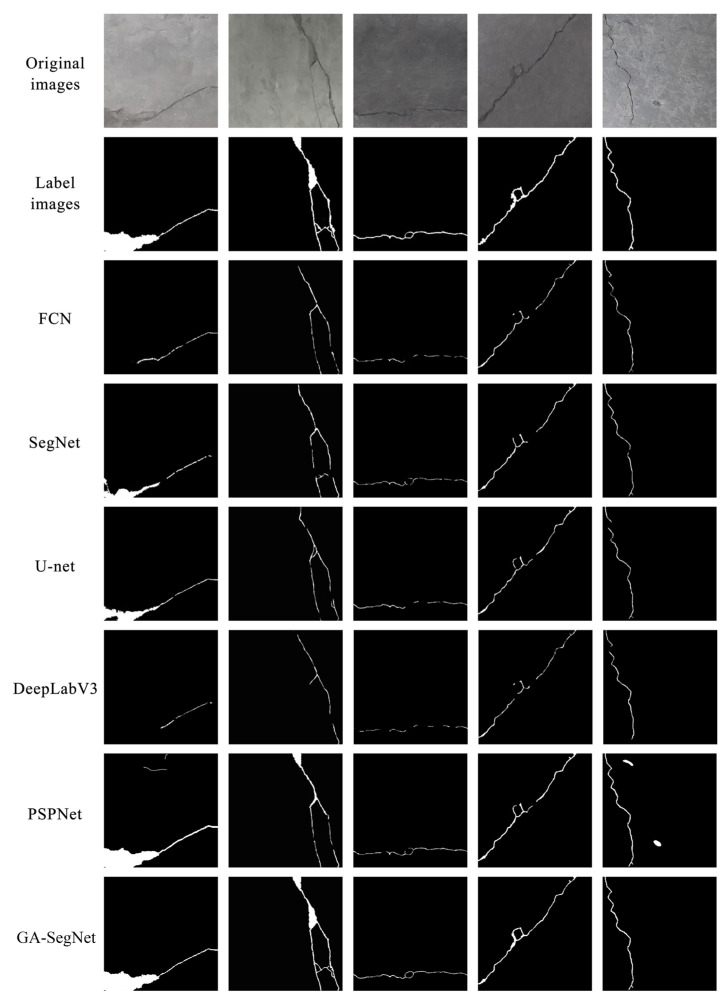
Effect picture of crack segmentation of GA-SegNet and reference model.

Figure 11 shows the crack segmentation results of different algorithms. It can be observed that DeepLab V3 had the poorest performance in crack segmentation, only extracting partial cracks and exhibiting poor matching of local shapes. It is not suitable for crack image segmentation in complex scenes. SegNet and U-Net had similar segmentation results, but SegNet performed better in terms of local details. FCN could segment wider cracks with less surrounding noise but failed to consider the intrinsic relationship between low-level and high-level features, resulting in poor segmentation performance for subtle cracks and edge processing. PSPNet showed good overall segmentation performance but performed relatively worse in handling noise in the image background. Compared to other reference models, the proposed GA-SegNet segmentation model in this study achieved closer prediction of crack integrity and real regions on the dataset, with lower output noise and more accurate extraction of edge details.

Table 7 illustrates the experimental results obtained by the advanced methods mentioned above on the Crack500 dataset. The GA SegNet network proposed in this paper showed the best performance among all evaluation metrics, achieving 78.24% mPA and 83.51% mIoU. Compared with FCN, our method’s mPA and mIoU increased by 9.67% and 12.57%, respectively. Compared with the PSP Net with the best overall performance, GA SegNet had improved various evaluation indicators, with PA, mPA, and mIoU increasing by 2.21%, 1.85%, and 2.49%, respectively.

Table 8 presents the evaluation metrics of the GA-SegNet network and reference networks on the CCUIPC test set. From the data results, it can be seen that DeepLab V3 had the worst evaluation result on the test set, with an mIoU of only 76.67%. Nevertheless, its PA and mPA evaluation parameters were slightly higher than those of FCN, reaching 91.52% and 83.27%, respectively. U-Net, SegNet, and PSPNet performed well, achieving an mIoU above 81.97% and PA above 96.21%. The proposed GA-SegNet network in this article exhibited the best performance in all evaluation metrics, with a precision of 89.79%, recall of 84.64%, F1 score of 87.13%, PA of 98.69%, mPA of 89.52%, and mIoU of 85.73%.

In short, the overall experimental results indicate that our proposed GA-SegNet algorithm outperforms the aforementioned segmentation algorithms in terms of inference efficiency and segmentation accuracy for small and indistinct crack targets in urban underground pipe corridors.

## 5. Conclusions

In this study, a semantic segmentation network based on global attention (GA-SegNet) is constructed. The GA-SegNet fully utilizes global contextual information and features at different scales to achieve fast and accurate crack segmentation. A residual separable convolution attention model is employed to extract features at multiple scales and achieve precise pixel classification in the image. A global attention model is utilized to enhance the network’s attention to crack regions and strengthen the connection between the encoder and decoder. High-dimensional features could guide low-dimensional features in a simpler way through weighted fusion, enabling the network to quickly and effectively adapt to features at different scales and improve the segmentation accuracy of crack regions. Finally, the impact of different loss functions on the performance of GA-SegNet is analyzed on the test set. Comparative experiments are conducted with classical semantic segmentation networks. The experimental results demonstrate that the proposed GA-SegNet model outperforms other models in terms of actual segmentation performance and various evaluation metrics on the dataset. Due to the high complexity and computational effort of the segmentation algorithm models, it is still more difficult to deploy them in embedded devices with fixed performance and limited computational resources. In the future, lightweighting the network to further balance efficiency and accuracy will be the focus of our research.

## Figures and Tables

**Table 1:** Detailed allocation table for Crack500 dataset.

	Training	Validation	Test	Total
cracks	1144	380	380	3428

**Table 2:** Detailed allocation table for CCUIPC dataset.

	Training	Validation	Test	Total
Pipe gallery cracks	1600	200	200	2000

**Table 3:** Experimental hardware configuration parameters.

Hardware	Configuration Parameter
Processor	Inter core i7-3770
GPU	NVIDIA GeForce GTX 1050 Ti
Memory	8 GB random access memory, 4 GB VRAM

**Table 4:** Experimental software configuration parameters.

Software	Configuration Parameter
Operating system	Windows 10
Programming Language	Python 3.7.15
Deep learning framework	PyTorch 1.2.0
CUDA	10.0
CUDNN	10.0
Anaconda	3-2021.04

**Table 5:** Results of ablation experiment.

Methods	RSCAM	GAM	WCE Loss	Dice Loss	Focal Loss	PA (%)	mPA (%)	mIoU (%)
FCN						91.41	81.93	79.84
Method (1)	√		√			93.68	82.92	78.64
Method (2)		√	√			94.73	83.06	80.95
Method (3)	√	√	√			95.65	83.81	81.62
Method (4)	√	√		√		97.31	87.21	82.37
Method (5)	√	√			√	98.69	89.52	85.73

**Table 6:** Model efficiency comparison.

Model	FPS	FLOPs (G)
FCN	30.3	94.5
SegNet	16.7	286
U-Net	38.6	104.0
Deep Lab V3	14.4	218.0
PSP Net	42.2	187.1
GA-SegNet	47	87.6

**Table 7:** Indicator parameters of GA-SegNet and reference model in Crack500 dataset.

Model	PA (%)	mPA (%)	mIoU (%)
FCN	78.25	68.57	70.94
SegNet	85.04	71.15	74.16
U-Net	87.21	73.94	72.85
Deep Lab V3	80.57	70.48	68.18
PSP Net	86.82	76.39	81.02
GA-SegNet	89.03	78.24	83.51

**Table 8:** Indicator parameters of GA-SegNet and reference model in CCUIPC dataset.

Model	PA (%)	mPA (%)	mIoU (%)
FCN	91.41	81.93	79.84
SegNet	96.21	85.44	83.27
U-Net	96.58	87.83	82.86
Deep Lab V3	91.52	83.27	76.67
PSP Net	97.31	86.95	81.97
GA-SegNet	98.69	89.52	85.73

## Data Availability

The data presented in this study are available on request from the corresponding author.
